# Design and preparation of novel domperidone loaded polymeric blend electrospun nanofibers for improved oral pharmacodynamic activity

**DOI:** 10.1007/s13346-025-01995-6

**Published:** 2025-11-06

**Authors:** Kamal Shatla, Eman Sweed, Suleiman Eltokhy, Adel Abdel-Rahman, Abdel Hamid Ismail, Nour Abd El-Sattar, El-Refaie Kenawy, Yusuf Haggag

**Affiliations:** 1 Department of Organic Chemistry, Faculty of Science, Menoufia University, Menoufia, Egypt; 2 Basic Medical Sciences Department, Faculty of Dentistry, Alryada University for Science & Technology, Sadat City, Egypt; 3 Department of Clinical Pharmacology, Faculty of Medicine, Menoufia University, Menoufia, Egypt; 4 Department of Clinical Pharmacology, Faculty of Medicine, Menoufia National University, Menoufia, Egypt; 5 Department of Pharmaceutical Technology, Faculty of Pharmacy, Tanta University, Tanta, 3111 Egypt; 6 Department of Chemistry, Faculty of Science, Ain Shams University, Abbassia, Cairo Egypt; 7 Department of Chemistry, Polymer Research Group, Faculty of Science, Tanta University, Tanta, Egypt

**Keywords:** Domperidone, Polymer blend, Nanofibers, Optimization, Prokinetic activity

## Abstract

Domperidone (DOM) is a BCS class II drug which widely used as an oral medication for the control of nausea and vomiting. It showed a very low and variable bioavailability related to its very poor solubility and pre-systemic metabolism. Polymeric nanofibers (PNFs) exhibit unique properties that make them a favorable choice for drug delivery applications. The study aimed to develop an optimum fast-dissolving oral polymeric electrospun nanofiber of DOM using Eudragit L100/Polyvinyl alcohol (EL-100/PVA) polymer blend. The fabrication process was optimized through a set of formulation parameters (composition of the polymer blend, polymer blend concentration, and electrospinning voltage). The DOM-loaded EL-100/PVA NFs were characterized for surface shape, nanosize, % drug loading, DOM in vitro release, drug/polymer interaction, and in vivo prokinetic study. The optimization process showed defect-free DOM-loaded NFs with very low nano diameter, high DOM loading, and superior in vitro dissolution of DOM at intestinal pH (about 90% of the drug released within 5 min). The drug/polymer interaction study demonstrated the conversion of DOM into an amorphous form, which facilitated its dissolution. No physicochemical interaction between DOM and the polymer blend was observed. I*n vivo* prokinetic study signified the orally enhanced prokinetic activity of DOM-loaded NF relative to pure DOM and commercial DOM product. The DOM-loaded EL-100/PVA NFs exhibited a better effect on the gastrointestinal reactivity relative to free DOM and commercial DOM product. The superior in vitro dissolution and in vivo prokinetic activity proved the promising potential of polymeric NFs to improve the oral delivery of DOM. The optimized DOM-loaded EL-100/PVA NFs may allow for dose reduction and low cardiovascular risk compared to conventional DOM tablets.

**Graphical abstract:**

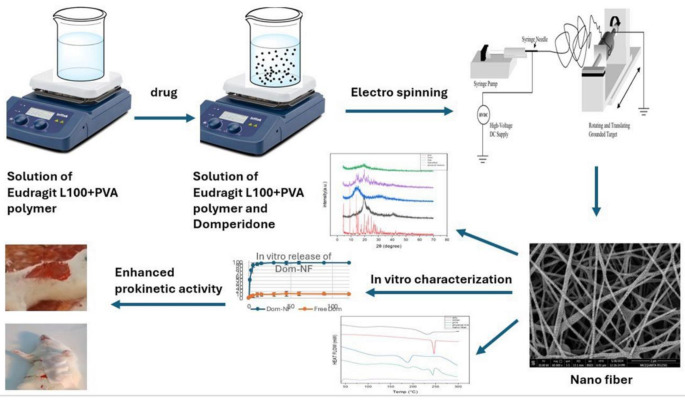

**Supplementary Information:**

The online version contains supplementary material available at 10.1007/s13346-025-01995-6.

## Introduction

Over the past few decades, controlled drug delivery systems (CDDs) have drawn significant attention as they have numerous benefits over traditional dosage forms. One of the most important benefits is the increased therapeutic efficacy and less in vivo toxicity via regulated delivery. Electrospun fibers have recently been investigated as a novel drug delivery technology for many drugs. The ability of fibrous carriers to deliver medications to different parts of the body is a unique advantage [1]. Additionally, more than one medication may be directly encapsulated within the fibers [2]. The very large surface areas, besides the porous nature of electrospun micro/nanofibers, make them useful in a variety of sectors, including photonics, tissue engineering, biosensors, drug-loaded membranes, nanocomposites, and other biomedical applications [3].

Amorphous solid dispersions containing fast-dissolving drug formulations are frequently created using electrospun nanofibers [4]. However, these nanofibers can also be employed in the creation of controlled-release and customized medications [5]. Tetracycline-loaded nanofibers prepared by Kenawy et al. used a polymeric combination of poly (lactic acid) (PLA) and polyethylene-co-vinyl acetate. This polymer blend maintained the tetracycline release for almost five days [6]. The production of nanofibers has recently shifted to electrospinning technology because of its ease of use, affordability, controllability, and diversity. It provides a practical, flexible, and efficient way to convert biobased raw materials into sophisticated nanomaterials and is an efficient way to produce nanofibers [7].

Formulation variables of the electrospinning technique can be categorized into three different groups, including parameters influencing the electrospinning process: ambient, process, and solution parameters. Each group of parameters has an impact on the fibers’ shape. Therefore, by optimizing these variables, electrospun micro/nanofibers with the required morphologies and diameters can be produced. Surface tension, conductivity, viscosity, and polymer content are some of the characteristics of the solution. The parameters of the process include the spinneret shape, feeding rate, voltage, and distance from tip to collector. Humidity, pressure, and temperature are all regarded as ambient parameters. The effects of these parameters in many studies have been thoroughly examined by our research group and other researchers [2, 8–10].

Different polymers are used to prepare drug-loaded oral polymeric electrospun nanofibers for tailored release. Eudragit L100 (EL-100) is an anionic copolymer comprising methyl methacrylate and methacrylic acid. EL-100 is commonly used as an enteric film coating. The active ingredients were protected from the stomach environment by the Eudragit L100 covering the enteric layer. Eudragit L100 showed pH-dependent solubility and developed strong molecular dispersion of amorphous drugs with enhanced physical stability [2, 8, 11, 12].

Polyvinyl alcohol (PVA) is a polymer having semi-crystalline, extremely hydrophilic, biodegradable, biocompatible, and nontoxic properties that have drawn a lot of attention because of its excellent pharmaceutical and biomedical applicability. These pharmaceutical properties include strong physical and thermal stability, water solubility, gas permeability, flexibility, and low cost. PVA is made by hydrolysis (metalizing) various grades of polyvinyl acetate (PVAc); the degree of hydrolysis (HD) determines the final properties of the used PVA polymer [13].

Domperidone (DOM) is an antiemetic drug that acts as a specific antagonist of both D2 and D3 receptors. It can be preferably used as a cure for nausea and vomiting accompanied by acute migraines; also, patients suffering from post-operative nausea and vomiting have been advised to use it [14]. DOM (BCS class II drug) is sparingly soluble in water, and its reported poor oral bioavailability ranged between 15 and 17% of the orally administered dose of 10 mg. This very low oral bioavailability originated from its high efflux mediated by small intestinal transporters, besides its low solubility in water, and extensive pre-systemic metabolism [15–18]. Different formulation techniques were reported to improve the in vitro dissolution of DOM using classical methods such as co-grinding, kneading, melt granulation, and co-evaporation and solid dispersion techniques [19–22]. The role of nanotechnology in improving the oral delivery of DOM was represented in very few research articles. Nanocrystals and solid lipid nanoparticles were investigated to boost the oral bioavailability of DOM [23–25]. Most recently, there has been only one research that reported the fabrication of DOM-Loaded NF in the form of an oral fast-dissolving film [16].

Polymeric electrospun micro/nanofibers are commonly used to prepare oral fast-dissolving solid dispersion formulations with amorphous drugs loaded into a tremendous surface area, ready for dissolution [26]. The use of two hydrophilic polymers of EL100 and PVA for preparing the DOM-loaded nanofibers was specifically intended to increase the dissolution rate of DOM as an insoluble drug. In this study, PVA was chosen for its superior hydrophilic nature, biocompatibility, and biodegradability, making it an ideal polymer for improving the solubility of DOM. On the other hand, EL100 was selected for its pH-sensitive profile, which enables controlled drug release at certain GIT locations, specifically the duodenum, where pH is elevated and drug is readily absorbed, thereby optimizing its therapeutic efficacy [27]. The integration of this polymer blend with the electrospinning technique will provide a scalable and clinically relevant drug delivery solution for Domperidone.

The objective of the provided study is to enhance the dissolution and aqueous solubility of DOM via the formulation of DOM-loaded EL100/PVA nanofibers. This hydrophilic polymer blend can produce drug-loaded NFs with a faster dissolution rate and onset of therapeutic activity, meanwhile minimizing the high variability related to DOM oral absorption. The formulation and physicochemical analyses of the produced DOM-loaded EL100/PVA nanofibers were modified using various process factors. The in vivo pharmacodynamic activity of the optimized DOM-loaded EL100/PVA NF was thoroughly investigated using experimental rats. As far as we are aware, this is the first study to describe delivering DOM using the EL100/PVA polymer combination for a better pharmacodynamic effect.

## Materials and methods

### Materials

Eudragit L-100 was a gifted sample from Evonik Healthcare, PVA (The average Mw is 72,000 g/mol with 98.9% hydrolysis), and was obtained from Biochemia, Germany. The pure reference standard of domperidone (DOM) was purchased from Sigma-Aldrich (USA). Ethanol, Disodium hydrogen phosphate, and Dipotassium hydrogen phosphate were purchased from Horus Laboratory Chemicals (Egypt). Deionized and double-distilled water were used throughout this work. The rest of the chemical ingredients were used without purification.

### Solution preparation for electrospinning

The proper amounts of polymer were dissolved in ethanol and heated gently until the polymer was completely dissolved to create a polymer solution of Eudragit L-100 using anhydrous ethanol. For two hours, the polymer was dissolved in a closed system with a magnetic stirrer. The DOM was added to the polymer solution using a magnetic stirrer till a homogenous mixture appeared. The PVA amount was dissolved in deionized water to prepare the PVA solution by heating using a hot magnetic stirrer for 3 h. The PVA solution was added gradually to the EL-100/DOM mixture and mixed for another 3 h for better homogeneity.

### Electrospinning

A syringe with a blunt-end needle and a ground electrode (a stainless steel sheet on a drum with a variable rotation speed), 20 cm from the needle’s end, comprised the setup for the electrospinning formulation technique used in this study (The Razel R99-E medical multiple speed syringe pump, Razel Scientific, USA). (NANON-01 A electrospinner, MECC CO., Japan) was used to prepare DOM-loaded NFs utilizing the electrospinning technology. The high voltage generator (HCP series, Fug Elektronik, Germany) was used to initiate the jet of the polymer solution with a minimal current output (of a few mA) by creating the voltage between the end of the needle and the tip of the collector. The polymer/drug solution received a positive voltage. The syringe pump supplied the prepared DOM/EL-100/PVA solution to regulate the feed rate, which was between 10 and 18 milliliters per hour. To create a thin sheet of non-woven nanofibers, the resultant nanofibers were gathered using a revolving copper drum. Table 1 lists the formulation and optimization parameters utilized to create the DOM-loaded NFs.

### In vitro characterization of DOM-loaded NFs

#### scanning electron microscopy (SEM) of DOM-loaded NFs

Using SEM equipment (NRC QUANTA FEG250), the electrostatically spun fibers’ surface morphology was evaluated. Nanofibers loaded with DOM and electrostatically spun were positioned in the center of an aluminum metal stud. The nanofibers from the sputtering unit were coated with a very thin layer of gold. After the prepared gold-coated fibers were placed inside the microscope chamber, the coated samples were immediately subjected to a high vacuum.

#### % drug loading (% DL) of DOM-loaded NFs

An aliquot of 10 mg of DOM-loaded NFs was thoroughly dissolved in 10 mL of dehydrated ethanol while being stirred magnetically for 5 h. The drug solution was analyzed using UV spectrophotometry at 284 nm using UV spectrophotometer equipment (UV 1800, Shimadzu, Japan) to concurrently quantify DOM in drug-loaded NFs [28]. A validated calibration curve was utilized to determine the drug concentration. The % DL for DOM was computed from equation no. (1) All prepared DOM-loaded NF samples were tested in triplicate.

1 $$ \begin{array}{l}{\rm{\% }}\,{\rm{EE}}\,{\rm{for}}\,{\rm{DOM}}\,{\rm{ = }}\,{\rm{The}}\,{\rm{amount}}\,{\rm{of}}\,{\rm{entrapped}}\,{\rm{DOM}}\,{\rm{/Total}}\,\\{\rm{added}}\,{\rm{amount}}\,{\rm{of}}\,{\rm{DOM}}\,{\rm{*100}}\end{array} $$

#### In vitro DOM release

An aliquot of free DOM, commercial DOM oral product (Gastromotil^®^, tablets, EPICIO, Egypt), and DOM-loaded NFs containing 10 mg of DOM was located in a tightly closed dialysis bag (MWCO is 12 KDa). The dialysis sac was immersed in 100 mL of a dissolution medium of phosphate buffer (pH 6.8). At predetermined time intervals, release samples (1 mL) were removed and replenished with fresh preheated phosphate buffer medium to keep the sink condition attained. Samples removed were filtered initially through a syringe (0.45-µm) and then analyzed at 284 nm using a validated UV spectrophotometric analysis reported earlier.

#### Fourier-transform infrared spectroscopy (FTIR) of DOM-loaded NFs

FTIR experiment was carried out on EL-100, PVA, DOM, DOM/EL100/PVA physical mixture, and DOM-loaded EL100/PVA NFs using an FTIR spectrophotometer (Tensor 27 Bruker, OPUs software, Billerica, MA, USA). The scanning range was recorded at ambient temperature and adjusted to be 4000–400 cm^− 1^ with a resolution of 4 cm^− 1^.

#### Differential scanning calorimetry (DSC) of DOM-loaded NFs

DSC experiment was performed on EL-100, PVA, DOM, DOM/EL100/PVA physical mixture, and DOM-loaded EL100/PVA NFs using the Mettler Toledo DSC1 STAR e instrument. Measurements were performed under an N_2_ atmosphere (10 mL/min) in the provided temperature range from 0 to 300 °C with a rate of heating of 20 °C/min.

#### X-ray diffraction (XRD) of DOM-loaded NFs

EL-100, PVA, DOM, DOM/EL100/PVA physical mixture, and DOM-loaded EL100/PVA NFs were all subjected to an X-ray diffractometer, K-ALPHA (Thermo Fisher Scientific, USA), for XRD investigation. The K-Alpha X-ray. The X-ray photoelectron spectroscopy data used in this investigation were collected using the photoelectron spectrometer system. The monochromatic radiation was produced with full-spectrum narrow-spectrum 50 eV and pass energy 200 eV at 10–9 mbar of pressure (10–1350 eV, 400 μm spot size).

### In vivo study

The in vivo study was conducted following ethical guidelines approved by Tanta University’s Animal Care & Use Committees and the National Institutes of Health’s protocol for the use and care of laboratory animals. Each procedure adhered to the previously set standards for the use and care of experimental animals that were approved by the CIOMS (TP/RE/1/25 P-01 is the ethical approval code).

Using statistics and a sample size selection program, the sample size of experimental animals was calculated to be fifty rats. This in vivo study has an 80% power level and a confidence level of 95%. Adult male Wistar rats weighing about 200–250 g were procured from the Tanta Center for Laboratory Animals, Tanta, Egypt. The animals were placed in five/cages under controlled daily conditions of atmospheric temperature, humidity, and the light cycle before and during the study at the welfare animal house of the Faculty of Medicine, Menoufia University. All rats were fed with standard pellet feed and water ad libitum. The selected animals were carefully treated based on all the guidelines prescribed by the ARRIVE criteria [29].

Rats were divided randomly into five groups of ten each based on the calculated average body weight.

#### Group 1 (Control)

served as the control that received distilled water daily by oral gavage at a dose of 10 mL/kg.

#### Group 2 (Disease) group

described below.

#### Group 3 (DOM)

received DOM at a dose of 10 mg/kg suspended in distilled water [30].

#### Group 4 (Commercial)

received Gasteromotil^®^ orally at a dose of 10 mg/kg suspended in distilled water.

#### Group 5 (DOM-loaded NFs) (F3)

received DOM-loaded NFs at the same dose of free drug suspended in distilled water.

Before receiving ketamine (100 mg/kg) and xylazine (4 mg/kg) anesthesia, rats in groups 2–5 were fasted for eighteen hours and only given free access to water. Following a midline laparotomy, a silk thread was used to tie the rat’s stomach fundus and pyloric sphincter. Six hours later, the rats were re-anesthetized, and the pyloric ligature was removed. The treatment regimen was continued for a total duration of seven days, after which, on day seven, the stomach and ileum were excised for further analysis [30].

#### In vivo prokinetic activity by the phenol red method

All rats were given a semi-solid test meal consisting of 2 mL of 1.5% methylcellulose solution with phenol red (Sigma Aldrich, USA) added at a dosage of 50 milligrams per 100 mL after all treatments had been administered to them for seven days. The prokinetic activity was investigated following the guidelines provided by [29–31]. By measuring the entire length of the rat’s small intestine and the total distance covered by phenol red, the gastrointestinal transit was calculated.

For calculating the percentage of gastric emptying, the stomach is removed and analyzed for residual marker content.

$$ \begin{array}{l}{\rm{Gastric}}\,{\rm{Emptying(\% )}}\,{\rm{ = (1}} - {\rm{Amount}}\,{\rm{of}}\,{\rm{marker}}\,{\rm{remaining}}\,\\{\rm{in}}\,{\rm{the}}\,{\rm{stomach/Total}}\,{\rm{amount}}\,{\rm{administered)}}\,{\rm{ \times 100}}\end{array} $$

#### In vivo isolated tissue experiments

The stomach and ileum were promptly removed, and fecal content was completely flushed with the cold Krebs–Ringer solution (pH 7.4) [31]. The rat’s stomach was opened from its greater curvature, and the ileum was subdivided into 1 cm sections. The stomach and the ileum tissues were pinned down in a flat direction on the dissecting dish. Following the mucosal removal, both the longitudinal muscle strips of the rat’s gastric fundus and the circular muscle strips of its ileum were mounted in 25 mL of organ baths containing the used Krebs–Ringer solution aerated with continuous airflow and kept warm at 37 °C. All segments were allowed to be equilibrated for 60 min while being longitudinally tensioned to 2 g. Every 15 min, the bathing solutions were replaced with a new Krebs solution. The resulting isometric force was monitored by an external force-displacement transducer using the software of Biopac MP36R (Biopac Systems, Goleta-CA, USA). The analysis of the recorded muscle contractions was performed with the Acknowledge 5 data acquisition software (Biopac Systems, USA). The used muscle strips from both the stomach and ileum were restored to their previous optimal point of length-tension relationship by treatment with 3 µmol/L acetylcholine (ACh). The tissues were then allowed to equilibrate for at least 1 h before experimentation, and the tissues were washed 4 times with an interval of 20 min (with a fresh Krebs solution preheated at 37 °C). After the state of equilibrium was attained, ACh was added to the organ bath fluid for about 30 s for each addition (10^− 7^-10^− 3^ M). The stomach and ileum muscle contractions were measured in gram-force units.

### Statistical analysis

The mean ± SD was used to reflect the in vitro and in vivo data. The normality of distribution of the data was assessed using the Shapiro-Wilk analysis test. Using GraphPad Prism software (GraphPad Software, MA, USA), one-way and two-way Analysis of Variances (ANOVA) were utilized to do statistical analysis of the various groups, which was followed by the post hoc Tukey’s test. A *p*-value of less than 0.05 is deemed significant.

## Results and discussion

The current study carefully examined how the properties of DOM-loaded NFs were impacted by three crucial process formulation variables, as indicated in Table 1. An optimization study was conducted using different polymer compositions, polymer concentrations, and applied voltage. Experimental physicochemical characteristics were studied, including fiber morphology, average diameter, % drug loading, and drug in vitro release.

**Table 1:** Formulation of DOM-loaded EL100/PVA NFs

Formula ID	Polymer Blend	Polymer Conc. (% w/v)	Voltage KV	Feed rate (mL/h)	Drug loading (% w/w)	Distance to the collector (cm)
F1	EL-100/PVA (70:30)	10	12	0.5	10	20
F2	EL-100/PVA (60:40)	10	12	0.5	10	20
F3	EL-100/PVA (50:50)	10	12	0.5	10	20
F4	EL-100/PVA (50:50)	8	12	0.5	10	20
F5	EL-100/PVA (50:50)	12	12	0.5	10	20
F6	EL-100/PVA (50:50)	10	10	0.5	10	20
F7	EL-100/PVA (50:50)	10	15	0.5	10	20

### Effect of polymer blend composition

**Fig. 1:**
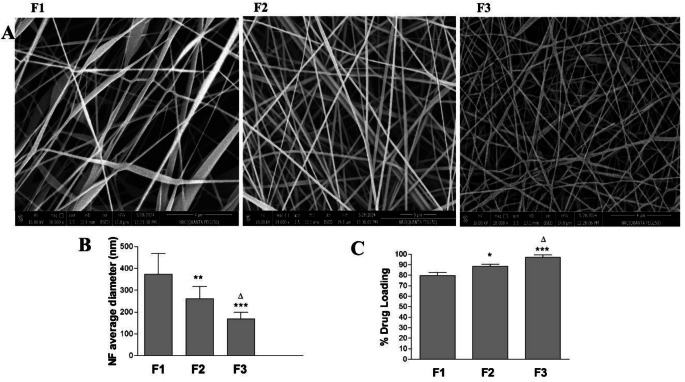
The effect of the composition of the polymer blend on (**A**) the SEM images, (**B**) average diameter, and (**C**) % drug loading of DOM-loaded NFs (F1, F2, and F3). Results are Mean ± SD & *n* = 3. *p < 0.05, **p < 0.01,***p < 0.001 compared with (F1). Δ*p* < 0.05, compared with (F2)

**Fig. 2:**
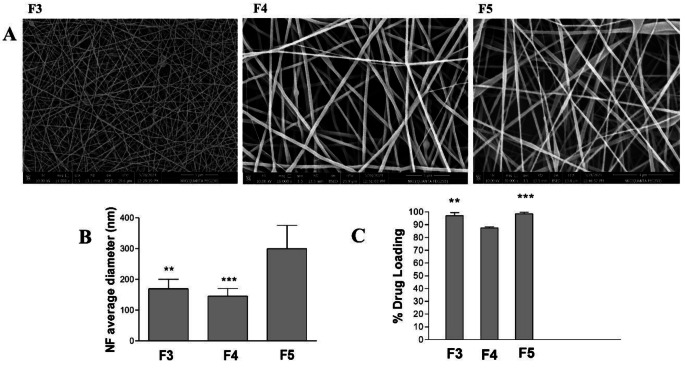
The effect of the concentration of the polymer blend on (**A**) the SEM images, (**B**) average diameter, and (**C**) % drug loading of DOM-loaded NFs (F3, F4, and F5). Results are Mean ± SD & *n* = 3. **p < 0.01,***p < 0.001 compared with (F5) in (B) and compared with (F4) in (C)

Figure 1 shows the effects of the polymer blend content on the physicochemical characteristics of the manufactured DOM-loaded NFs (F1, F2, and F3). The SEM pictures (Fig. 1.A), average NF sizes (Fig. 1.B), and percentage drug loading (Fig. 1.C) of three distinct formulations of F1, F2, and F3 are displayed in Fig. 1 and Supplementary Fig. 1. At a set electrospinning voltage of 12 KV and a fixed polymer blend concentration of 10% w/v, the impact of increasing the PVA concentration in the blend’s composition was examined. The EL-100/PVA polymer combination demonstrated a continuous, bead-like nanofiber with smooth surfaces that overlapped. All formulations’ surfaces (F1, F2, and F3) were devoid of visible particles. The size of DOM-loaded EL-100/PVA NFs (F1) with the lowest conc of 30% PVA recorded the highest average nanosize of 373 ± 95 nm. However, the average size of F2 and F3 was significantly lower compared to F1. The higher PVA content of 40% and 50% in the case of F2 and F3, respectively, resulted in smaller diameter nanofibers. The F3 with the highest concentration of PVA achieved a significantly smaller diameter of 196 ± 31 nm compared to F2. These findings are explained by the anticipated modifications in the polymer solutions’ conductivity and viscosity that occur when PVA concentration is increased while maintaining the same polymer concentration. The drug/polymer blend spinning solution becomes more conductive when the PVA concentration increases. The higher the electrical conductivity of the polymer blend solution, the smaller the NF diameters were obtained [32, 33]. Figure 2. C shows the % drug loading of (F1, F2, and F3). Increasing the PVA content resulted in a significant increase in % drug loading. F3 with the highest PVA concentration showed a % drug loading of 97 ± 2.5 compared to F2 and F1. This can be attributed to the higher solubility of the drug inside the PVA polymeric matrix, which can be reflected in better homogeneity and uniform distribution of DOM inside the hydrophilic matrix of the polymer blend. In order to create a uniform and transparent electrospinnable drug/polymer blend solution, the optimal solvent mixture of water/ethanol has shown high efficacy in dissolving both EL-100 and PVA polymers as well as DOM [35]. Adding additional hydrophilic polymers, like polyethylene oxide, to the Eudragit L-100 polymer produced comparable outcomes [2]. Increasing the PVA content to 60% showed DOM-loaded NFs full of beads with a very low content of nanofibers (Supp. Figure 2). The % drug loading of the prepared NFs with the highest PVA content of 60% showed a significant reduction in drug content of 83.9 ± 1.7. Increasing the PVA content to higher than 50% significantly increased the viscosity of the electrospinning solution. Using the same polymer concentration of 10% w/v, voltage of 12 KV, and feed rate of 0.5 mL/h resulted in an NF sample fully covered with beads with lower drug loading. This first optimization step showed that the optimum polymer composition that can be used for further optimization studies is (EL-100/PVA 50:50).

### Effect of the polymer blend’s concentration

The effect of the polymer blend’s concentration on the physicochemical properties of the prepared DOM-loaded NFs (F3, F4, and F5) is exhibited in Fig. 2 and Supplementary Fig. 3. Figure 2 shows the SEM images (Fig. 2.A), average NF sizes (Fig. 2.B), and % drug loading (Fig. 2.C) of F3, F4, and F5. The influence of changing the concentration of the polymer blend at a fixed polymer composition of (EL-100/PVA of 50:50) and fixed electrospinning voltage of 12 KV was demonstrated. The lowest polymer concentrations of 8% (F4) exhibited a non-significant reduction in the nanosize compared to F3. Notably, a heterogeneous distribution in the nanosize with irregular morphology of the produced nanofibers was observed. The average nano diameter of the generated NFs of (F5) increased noticeably when the concentration of the polymer blend was increased from 10% to 12% w/v. The mean diameter was greater at 299.5 ± 76 nm at the maximum polymer concentration of 12% w/v. As long as the conductivity of the polymer solutions of F3, F4, and F5 is the same and the polymer blend content is the same, that is. The viscosity of the (F5) polymer solution will rise as the concentration of the polymer blend increases. Because of the increased time required for stress relaxation at the same electrical field voltage, the EL-100/PVA polymer solution’s higher viscosity produced a fiber with a larger nanodiameter [32, 34].

The effect of the polymer blend’s concentration on the % drug loading of DOM is shown in (Fig. 2.C). The % drug loading for DOM was high for both formulations (F3 and F5) with a polymer blend’s concentration of 10 and 12% w/v, compared to F4 with a lower polymer concentration of 8% w/v. The lower concentration of the polymer blend resulted in lower viscosity of the hydrophilic polymeric solution, which might have led to certain drug precipitation in the collector part and thereby decreased the amount of DOM loaded into the prepared NFs [35]. On the other hand, higher polymer blend concentrations of 10 and 12% w/v improved the solubility of DOM in the hydrophilic polymeric solution and enhanced drug loading inside the matrix of the polymer blend [8]. This second step of optimization proved that the best polymer concentration is 10% w/v, which will be used for further optimization studies.

### Effect of electrospinning voltage

**Fig. 3:**
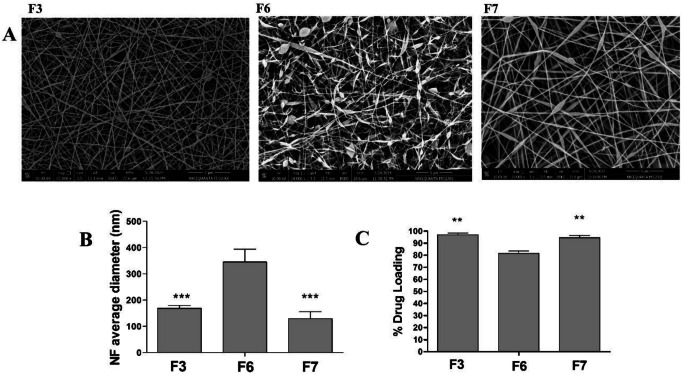
The effect of the electrospinning voltage on (**A**) the SEM images, (**B**) average diameter, and (**C**) % drug loading of DOM-loaded NFs (F3, F6, and F7). Results are Mean ± SD & *n* = 3. **p < 0.01,***p < 0.001 compared with (F6)

The influence of the electrospinning voltage applied on the physicochemical behavior of the prepared DOM-loaded NFs (F3, F6, and F7) is exhibited in Fig. 3 and Supplementary Fig. 4. Figure 3 shows the SEM images (Fig. 3.A), average NF sizes (Fig. 3.B), and % drug loading (Fig. 3.C) of F3, F4, and F5. The impact of changing the voltage values while fixing the polymer composition of (EL-100/PVA of 50:50) and polymer concentration of 10% was studied. Decreasing the applied voltage from the middle level of 12 KV to the lowest level of 10 KV showed a significant deformation of the NF shape, represented by irregular shapes of the NFs with a lot of beads covering the fibers. The significant increase in the NF diameter of F6 produced at lower voltage is highly proportional to the formation of beads. The formed NF’s nano diameter decreased non-significantly when the voltage was increased from 10 KV (F3) to 15 KV (F7). DOM-loaded EL-100/PVA NFs (F7) made at the maximum voltage of 15 KV also displayed some beads, but with lesser diameters. The critical function of choosing the ideal value of the exposed electrical voltage was demonstrated by the noticeable rise in the average nanosize of the F6 drug-loaded nanofibers by lowering the electrospinning voltage. A smaller diameter of NFs was obtained by increasing the spinning voltage since it increased the rate of solvent evaporation and maximized the charge repulsion within the jet [36, 37].

The impact of process voltage on the % drug loading of DOM is shown in (Fig. 3.C). The % drug loading for DOM was significantly higher for both formulations (F3 and F7) with applied voltage of 10 and 15 KV compared to F6 with the lowest voltage of 10 KV. The lower voltage resulted in gradual drug precipitation out of the polymer solution and consequently decreased the amount of DOM loaded into the prepared NFs [2, 37]. The previous results showed that DOM-loaded EL-100/PVA NFs (F3) were the optimum formula as they exhibited superior physicochemical characteristics regarding morphology, average diameter, and DOM loading compared to the rest of the formulas.

DOM-loaded EL-100/PVA NFs (F3) were selected for further in vitro investigations and in vivo evaluations.

### In vitro DOM release

**Fig. 4:**
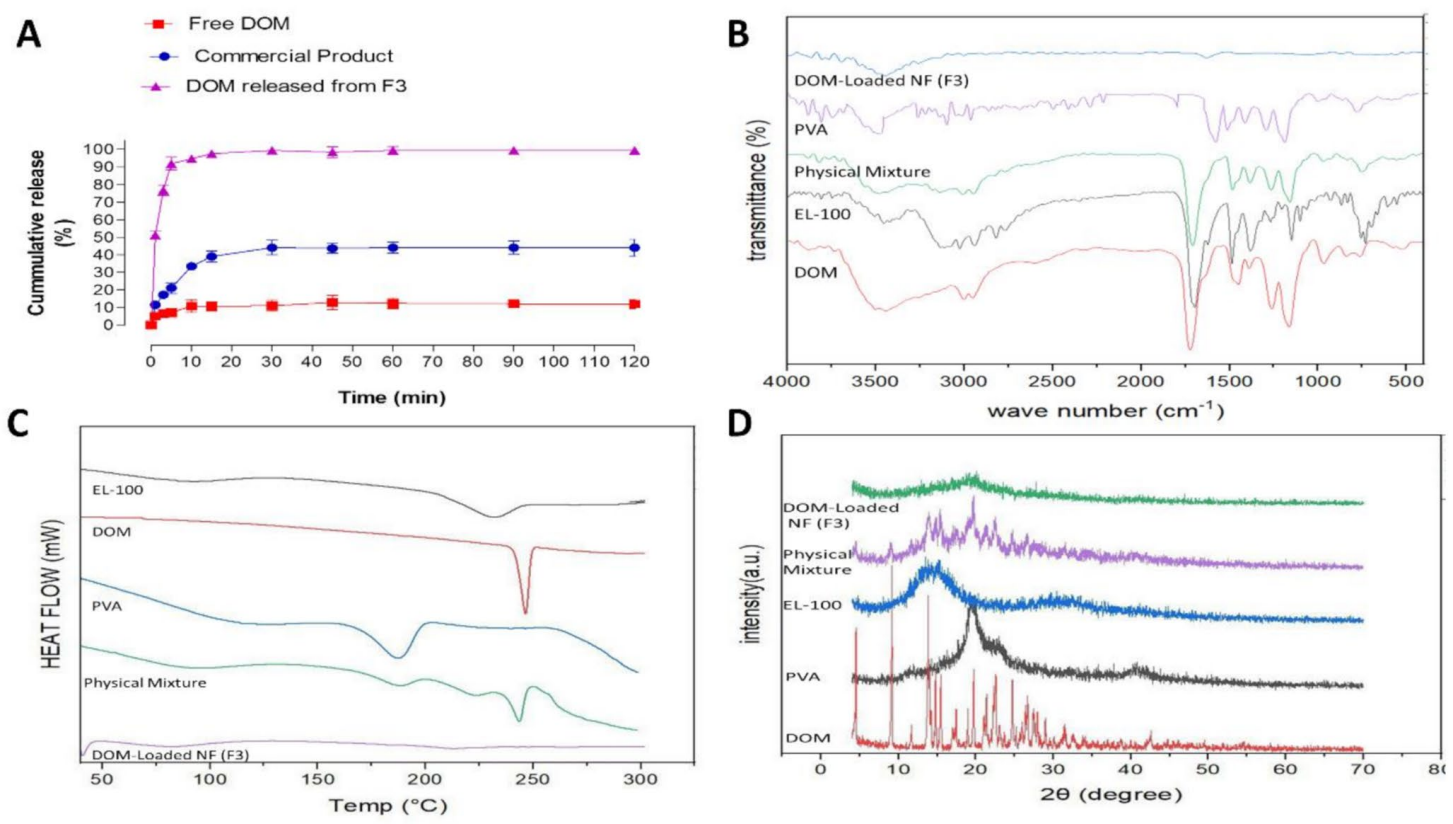
The in vitro characterization of DOM-loaded NFs, in vitro release of optimum DOM-loaded NFs (F3), commercial DOM product, and free DOM at a pH of 6.8, results are Mean ± SD & *n* = 3 (**A**). FTIR spectra for DOM, EL-100, PVA, physical mixture, and DOM-loaded NFs (F3) (**B**). DSC thermograms for DOM, EL-100, PVA, physical mixture, and DOM-loaded NFs (F3) (**C**), and XRD spectra for DOM, EL-100, PVA, physical mixture, and DOM-loaded NFs (F3) (**D**)

The in vitro release of DOM from commercial product (Gastromotil^®^) and DOM-loaded EL-100/PVA NFs (F3) is illustrated in Fig. 4.A. The release figures for other DOM-loaded EL-100/PVA NFs were represented in Supplementary figures (4–7). Free DOM showed very low dissolution at a pH of 6.8. The total amount of drug dissolved after 120 min is about 11.97 ± 4.23. The commercial product showed higher in vitro release compared to free DOM. Approximately 21.09% of DOM was released within 5 min of the commercial product; meanwhile, a total amount of 44.1% was released after 2 hours of in vitro release. However, DOM-loaded EL-100/PVA NFs (F3) showed a significantly higher in vitro release of over 90% released within the first 5 min. The addition of PVA to EL-100 showed a significantly higher release of DOM from F3 compared to the free drug and the drug released from the commercial product. Our results showed that EL-100/PVA can efficiently release DOM at an intestinal pH of 6.8, where DOM is readily absorbed from the duodenum part which can greatly improve its oral bioavailability [38, 39]. The hydrophilic nature of EL-100/PVA accounts for its potent wetting capacity, which speeds up the DOM release from the NFs. Additionally, the F3 has a huge surface area and substantial porosity, which, when combined, improves the contact with dissolution media and hence promotes drug release. Similar results were reported about the release of poorly soluble drugs from the Eudragit L100/PEO polymeric mixture [2]. The in vitro release kinetics were thoroughly studied, and the data of the release fitting and the correlation coefficient values (R^2^) are provided in Supplementary Table 1. The release data was fitted to various release kinetic models (Zero, First, Higuchi, and Korsmeyer-Peppas). All DOM-loaded EL-100/PVA NFs followed the Korsmeyer-Peppas kinetic model. An identical kinetic model was proven by previously prepared nanofibers using hydrophilic polymers encapsulating poorly soluble drugs [2]. Release exponent (n) was less than 0.5 in all prepared formulations, which indicates Quasi-Fickian diffusion with a non-swellable matrix-diffusion drug release strategy. The release of DOM from the monolithic matrix is diffusion-controlled.

The large surface-to-volume ratio, specific surface area, and high porosity of the microporous structure of nanofiber networks are advantageous for higher encapsulation and direct incorporation of poorly soluble drugs such as DOM, leading to an improved in vitro dissolution and release performance [40, 41]. To further increase the DOM solubility using these nanofibrous structures, a blend of two hydrophilic polymers was adopted to enhance its in vivo absorption. DOM is rapidly absorbed from the GIT, and its serum level’s peak appears within 30 min. DOM showed a rapid onset of action, typically within 1 h of its oral administration [42–44]. DOM-loaded EL-100/PVA NFs (F3) resulted in 90% of DOM released after 5 min, which will facilitate the rapid drug absorption from the GIT and hence its onset of action without any possible risk of drug accumulation inside the GIT.

### FTIR analysis

The FTIR spectra of pure DOM, PVA, EL-100, DOM/EL-100/PVA physical mixture, and DOM-loaded EL-100/PVA NFs (F3) are shown in Fig. 4.B. The possible interaction between DOM and EL-100/PVA polymers was investigated by the FTIR study. The presence of any modifications in the molecular bonds between specific functional structural groups is used to illustrate the absence of a drug crystal structure. The pure DOM showed a set of unique absorption peaks at 1,346 cm^− 1^ of C-N amine stretching, 1,400–1,600 cm^− 1^ for C-H aromatic stretching, and 1,697 cm^− 1^ for the N-C = O amide stretching. The polymer EL-100 spectrum includes two bands at 1175 cm^− 1^, 1270 cm^− 1,^ and a sharp band at 1735 cm^− 1^ caused by ester linkages of the (C–O), a band between 2200 and 3400 cm^− 1^ for the (O–H) stretch vibration. PVA was found to have its primary peaks at 839, 1081, 1425, 1690, 2917, and 3280 cm^− 1^. The peaks of PVA are attributed to the following: C-H asymmetric stretching vibration, deformation vibration, and bending vibration of CH 2, C–C stretching vibration, C–O stretching of acetyl groups, O–H stretching vibration, and C = O carbonyl stretch. The physical mixture showed all peaks of the DOM and EL-100/PVA polymer blend. The absence of any interaction between DOM and the EL-100/PVA polymers used for NF formulation was confirmed by the FTIR spectra of the DOM/EL-100/PVA physical mixture and DOM-loaded NFs (F3), which displayed spectral characteristics comparable to those of pure DOM. These observations strongly demonstrated the existence of any chemical or physical interactions between the DOM and other EL-100/PVA polymers used during NF preparation.

### DSC analysis

The DSC thermograms of pure DOM, PVA, EL-100, DOM/EL-100/PVA physical mixture, and DOM-loaded EL-100/PVA NFs (F3) are shown in Fig. 4.C. DSC analysis was employed in this investigation to evaluate the components’ physical states and possible drug-polymer interactions. The EL-100 polymer’s scan showed a broad dehydration endothermic band below 100 °C because of its amorphous condition, followed by a broad endotherm over 200 °C because of the degradation of the polymer. The melting point of free DOM is indicated by the thermogram’s sharp endothermic peak at 242.5 ^◦^C. The thermogram of PVA showed a relatively broad endothermic peak at 184.3 ^◦^C, which indicates the possible dehydration of the polymer. Compared to crystalline polymers, amorphous polymers, such as PVA, are more prone to moisture sorption [45]. The DOM/EL-100/PVA physical mixture showed all peaks of the DOM and polymer blend. In the case of DOM-loaded EL-100/PVA NFs (F3), the drug’s sharp endothermic peak completely disappeared from the thermogram, confirming the complete drug’s dispersion inside the EL-100/PVA matrix and leading to the amorphous conversion of the drug in the NF.

### XRD analysis

The XRD study was carried out on pure DOM, PVA, EL-100, DOM/EL-100/PVA physical mixture, and DOM-loaded EL-100/PVA NFs (F3), and the results are highlighted in Fig. 4.D. The X-ray diffraction spectrum of the free DOM showed the presence of multiple sharp, distinct peaks, which confirm the crystalline arrangement of the pure drug represented by the characteristic diffraction pattern. EL100 and PVA diffraction spectra showed no sharp characteristic peaks, which indicated the amorphous nature of the polymer blend. The physical mixture shows all the characteristic sharp diffraction peaks of DOM particles, with reduced intensity, indicating that DOM is keeping its crystalline state inside the physical mixture. In contrast, the XRD pattern of DOM-loaded EL-100/PVA NFs (F3) showed no peaks of crystalline DOM, indicating that all the DOM loaded into the EL/PVA NFs no longer existed as crystalline moieties but had completely transformed into an amorphous state. These results augmented the FTIR and DSC results, which proved the full transformation of the DOM from the crystalline to the amorphous nature. DOM molecules were highly dispersed inside the EL-100/PVA nanofiber matrix, and hence their crystalline nature was lost.

The DOM-loaded into EL-100/PVA NFs (F3) showed less intense and highly diffused peaks of DOM, which indicates the reduced ordering of DOM’s crystal lattice, confirming the complete formation of the amorphous solid state of this drug [46]. The formation of amorphous solid dispersion is intended through nanofiber formulations to take advantage of the higher apparent amorphous solubility, which increases the dissolution of poorly water-soluble drugs such as DOM [16]. The extent of a drug’s crystallinity influences its dissolution, especially for poorly water-soluble drugs. DOM-loaded EL-100/PVA NFs showed DOM in a less crystalline and an amorphous form compared to pure DOM. Amorphous DOM dissolves at a faster rate because of greater molecular motion and higher internal energy, which enhances the thermodynamic properties of DOM as compared to its crystalline form [47]. Nanoformulation was reported to improve the in vitro dissolution and in vivo delivery of other drugs through different nano-drug delivery approaches [48–50].

### In vivo prokinetic activity

Domperidone is not only used for the treatment of GIT disorders such as nausea and vomiting, but also used as adjunctive treatment for other diseases such as diabetes for the management of diabetic gastroparesis [51, 52]. Repurposing of Domperidone has also been reported for the treatment of multiple sclerosis and colorectal cancer [53–55].

#### Evaluation of prokinetic activity by phenol red method

**Fig. 5:**
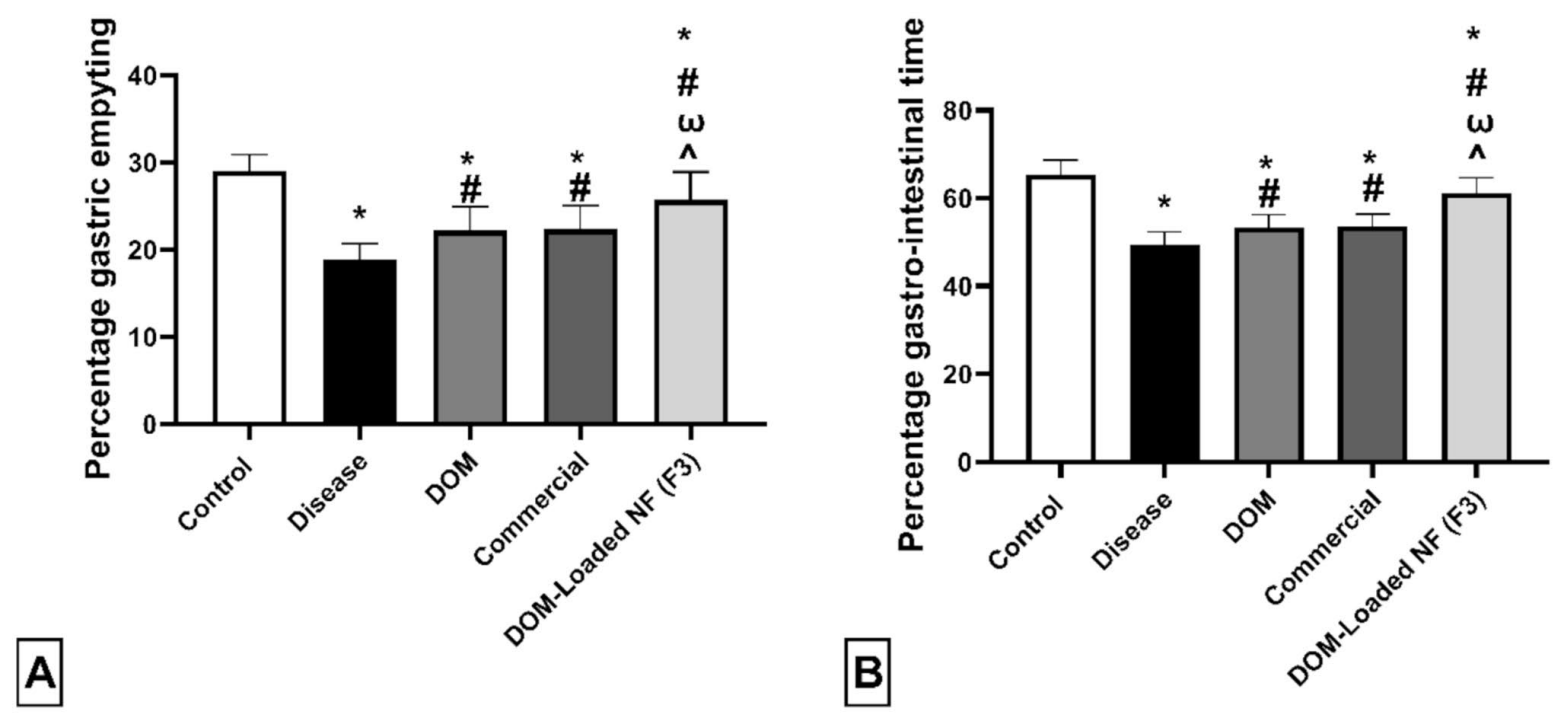
Effect of DOM, commercial DOM product, and DOM-loaded NFs (F3) on prokinetic activity. (**A**) percentage gastric emptying and (**B**) percentage gastrointestinal transit. Data were presented as mean ± SD. Data were compared using a one-way ANOVA test (*n* = 10). * compared to the control group, ^#^ compared to the disease group, and ^Ѡ^ compared to the DOM group, ^ compared to the commercial group. DOM: domperidone, and DOM-loaded NFs: domperidone-loaded nanofibers (*p* < 0.05)

Rats in the disease group had significantly lower values (*p* < 0.05) for both gastrointestinal transit and gastric emptying percentage than rats in the control group (18.85 ± 1.84 vs. 29.00 ± 1.87 and 49.45 ± 2.93 vs. 65.35 ± 3.31). Rats in the DOM, Commercial, and DOM-loaded NFs (F3) groups showed significantly higher gastric emptying (22.20 ± 2.75, 22.35 ± 2.69, and 25.40 ± 3.57) and gastrointestinal transit (53.45 ± 2.82, 53.55 ± 2.80, and 61.25 ± 3.43) than rats in the control and illness groups (*p* < 0.05). Additionally, the percentage of gastrointestinal transit and stomach emptying in the DOM-loaded NFs (F3) group was significantly higher (*p* < 0.05) than in the DOM and Commercial groups (Fig. 5).

#### Evaluation of ileal and stomach fundus contraction

**Fig. 6:**
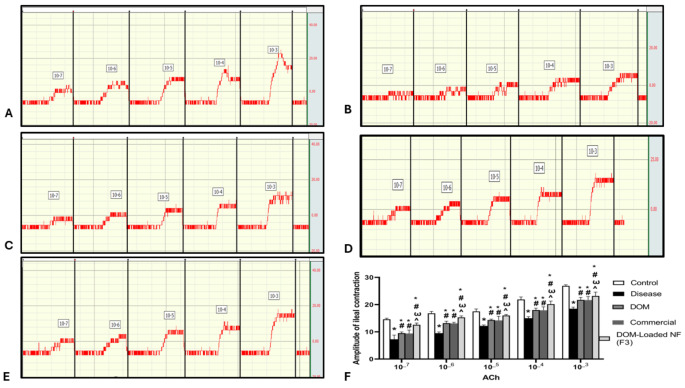
Effect of DOM, commercial DOM product, and DOM-loaded NFs (F3) on amplitude of ileal contraction. (**A**) control, (**B**) disease, (**C**) DOM, (**D**) Commercial, (**E**) DOM-loaded NFs (F3), and (**F**) statistical analysis. Data were presented as mean ± SD. Data were compared using a two-way ANOVA test (*n* = 10). * compared to the control group, ^#^ compared to the disease group, ^Ѡ^ compared to the DOM group, ^ compared to commercial. DOM: domperidone, and DOM-loaded NFs: domperidone-loaded nanofibers (*p* < 0.05)

**Fig. 7:**
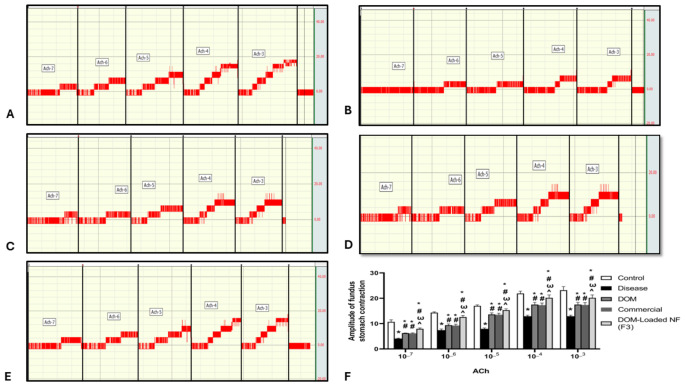
Effect of DOM, commercial DOM product, and DOM-loaded NFs (F3) on amplitude of stomach fundus contraction. (**A**) control, (**B**) disease, (**C**) DOM, (**D**) Commercial, (**E**) DOM-loaded NFs (F3), and (**F**) statistical analysis. Data were presented as mean ± SD. Data were compared using a two-way ANOVA test (*n* = 10). * compared to the control group, ^#^ compared to the disease group, ^Ѡ^ compared to the DOM group, ^ compared to commercial. DOM: domperidone, and DOM-loaded NFs: domperidone-loaded nanofibers (*p* < 0.05)

Exposing the isolated ileum and stomach fundus to ACh resulted in a significantly reduced amplitude of the rhythmic contractions in the disease group compared to the control group (*p* < 0.05). While in the DOM, Commercial, and DOM-loaded NFs (F3) groups, there was a significant increase in the amplitude of rhythmic contractions after exposure of isolated ileum and stomach fundus to ACh (*p* < 0.05). Moreover, there was a marked increase in the amplitude of rhythmic contractions in the DOM-loaded NFs (F3) group compared to the DOM and Commercial groups (*p* < 0.05) (Table 2; Figs. 6 and 7). The data presented in Table 2 are also represented graphically in Figs. 6-F and 7-F to facilitate clearer visualization and comparison of the contraction responses among treated groups.

**Table 2:** Effects of DOM, commercial DOM product, and DOM-loaded NFs (F3) on the amplitude of ileal and stomach fundus contraction

	Control	Disease	DOM	Commercial DOM Product	DOM-loaded NFs (F3)
ACh effect on the ileum					
10^_7^	14.60 ± 0.40	7.25 ± 1.56*	9.50 ± 0.45*^#^	9.40 ± 1.26*^#^	12.60 ± 0.64*^#Ѡ^^
10^_6^	16.80 ± 0.70	9.50 ± 0.45*	13.20 ± 0.70*^#^	13.10 ± 0.43*^#^	15.30 ± 0.56*^#Ѡ^^
10^_5^	17.50 ± 0.90	12.20 ± 0.32*	14.33 ± 0.32*^#^	14.22 ± 1.52*^#^	16.00 ± 0.35*^#Ѡ^^
10^_4^	21.90 ± 0.91	15.00 ± 0.55*	18.00 ± 0.70*^#^	17.95 ± 1.24*^#^	20.20 ± 1.11*^#Ѡ^^
10^_3^	26.90 ± 0.40	18.50 ± 0.58*	21.73 ± 0.91*^#^	21.70 ± 1.25*^#^	23.20 ± 1.40*^#Ѡ^^
ACh effect on the stomach fundus					
10^_7^	10.70 ± 0.80	4.09 ± 0.16*	6.24 ± 0.07*^#^	6.15 ± 0.20*^#^	7.94 ± 0.33*^#Ѡ^^
10^_6^	14.33 ± 0.32	7.42 ± 0.47*	9.37 ± 0.49*^#^	9.26 ± 0.49*^#^	12.60 ± 0.64*^#Ѡ^^
10^_5^	17.00 ± 0.40	7.94 ± 0.33*	13.60 ± 0.67*^#^	13.50 ± 0.62*^#^	15.30 ± 0.56*^#Ѡ^^
10^_4^	21.90 ± 0.91	12.90 ± 0.43*	17.50 ± 0.90*^#^	17.30 ± 0.81*^#^	20.20 ± 1.11*^#Ѡ^^
10^_3^	23.20 ± 1.40	12.90 ± 0.43*	17.50 ± 0.90*^#^	17.40 ± 0.91*^#^	20.20 ± 1.11*^#Ѡ^^

The data is presented as mean ± standard deviation (*n* = 10). Statistically compared using the two-way ANOVA test; * compared to a control group, # compared to the disease group, Ѡ compared to the DOM group, and ^ compared to commercial. ACh: acetylcholine, DOM: Domperidone, and NFs: Nanofibers.

In this study, DOM, Commercial, and DOM-loaded NFs (F3) increased the power of gastric emptying and significantly elevated the percentage of gastrointestinal transit compared to the disease group, with better results in the group administered with DOM-loaded NFs (F3). This follows what was reported by Jang et al. [56]. The rats treated with DOM-loaded NFs (F3) showed a better effect on the GIT reactivity compared to DOM and commercial groups. DOM is well-known to have anti-cholinesterase activity [57]. Based on the findings of that study, we assumed that DOM-loaded NFs (F3) would absorb better to give a better effect than free DOM or a commercial product. The optimized nanofiber formulation allows for dose reduction compared to conventional DOM tablets. The possible dose reduction of DOM-loaded NFs has a low risk of adverse cardiovascular events while exhibiting good clinical efficacy. The significantly improved prokinetic activity of the DOM-loaded NFs, without any observed adverse effects or mortality during the 7-day study period, underscores their potential therapeutic advantage. It is important to note that this study was designed as a proof-of-concept to demonstrate enhanced efficacy. While the formulation components (Domperidone, Eudragit L100, and PVA) are all pharmaceutically approved and possess favorable safety profiles [58–60]. The DOM dose used in vivo was proved to be clinically safe in experimental rats [18, 61]. A comprehensive long-term toxicological study falls outside the scope of this initial investigation. Future work will include detailed safety, pharmacokinetic, and biodistribution studies to fully validate the clinical potential of this nanofiber formulation.

## Conclusions

Electrospinning is a versatile technique commonly used to prepare polymeric nanofibers for drug delivery purposes. The impact of different process variables of electrospinning on average fiber diameter, surface uniformity, and total fiber quality was explored. The selection of the optimum composition of the EL-100/PVA polymer blend, polymer concentration, and spinning voltage successfully produced long, continuous DOM-loaded NFs (F3) with a smaller average diameter of less than 200 nm and high drug loading of approximately 97%. In vitro DOM release indicated that optimum DOM-loaded NFs were superior to the pure drug and commercial DOM with more than 90% of DOM released from (F3) within 5 min. The transformation of the crystalline behavior of DOM into the amorphous form after nanofiber formation using a hydrophilic polymer blend of EL100/PVA signified their role in marked dissolution enhancement. The higher DOM dissolution resulted in an improved pharmacodynamic activity in experimental animals. A significant improvement in the prokinetic activity of the DOM-Loaded NFs compared to pure DOM suspension and commercial DOM was observed in vivo. In conclusion, DOM-loaded EL-100/PVA NF can be effectively used to improve the DOM’s in vitro aqueous solubility and dissolution, meanwhile enhancing its pharmacological activity after oral delivery, in vivo. The possible dose reduction of DOM-loaded NFs will have a low risk of adverse cardiovascular events while exhibiting good clinical efficacy. Further investigations are required to explain the oral bioavailability and long-term safety of drug-loaded NFs to support their clinical translation to humans.

## Supplementary Information

Below is the link to the electronic supplementary material.

Supplementary Material 1

## Data Availability

Data will be available upon request.
