# Supplementary material for: Design and preparation of novel domperidone loaded polymeric blend electrospun nanofibers for improved oral pharmacodynamic activity
Source: Drug Deliv Transl Res. 2025 Nov 6;16(6):1966–80. doi: 10.1007/s13346-025-01995-6 (PMC13183739; doi:10.1007/s13346-025-01995-6)
Supplement: Supplementary file 1 — Supplementary Material 1 [file 13346_2025_1995_MOESM1_ESM.docx]

**Supplementary Data**

**Design and Preparation of Novel Domperidone Loaded Polymeric Blend Electrospun Nanofibers for Improved Oral Pharmacodynamic Activity.**

Kamal Shatla^1,2^, Eman Sweed^3,4^, Suleiman Eltokhy^5^, Adel Abdel-Rahman^1^, Abdel Hamid Ismail^1^, Nour Abd El-Sattar^2,6^, El-Refaie Kenawy^7^, Yusuf Haggag ^5^ ⃰

^1^ Department of Organic Chemistry, Faculty of Science, Menoufia University, Menoufia, Egypt.

^2^ Basic Medical Sciences Department, Faculty of Dentistry, Alryada University for Science & Technology, Sadat City, Egypt.

^3^ Department of Clinical Pharmacology, Faculty of Medicine, Menoufia University, Menoufia, Egypt.

^4^ Department of Clinical Pharmacology, Faculty of Medicine, Menoufia National University, Menoufia, Egypt.

^5^ Department of Pharmaceutical Technology, Faculty of Pharmacy, Tanta University, Tanta, Egypt.

^6^ Department of Chemistry, Faculty of Science, Ain Shams University, Abbassia, Cairo, Egypt.

^7^ Department of Chemistry, Polymer Research Group, Faculty of Science, Tanta University, Tanta, Egypt.

**⃰ Corresponding Author:**

Yusuf Haggag, Ph.D, AFHE

Associate Professor,

Department of Pharmaceutical Technology,

Faculty of Pharmacy, Tanta University,

Tanta 3111, Egypt.

Tel: +2 01220104612

Email: [youssif.hagag@pharm.tanta.edu.eg](mailto:youssif.hagag@pharm.tanta.edu.eg)

ORCID ID: <https://orcid.org/0000-0002-6203-1713>

Scopus : <https://www.scopus.com/authid/detail.uri?authorId=56800697500>


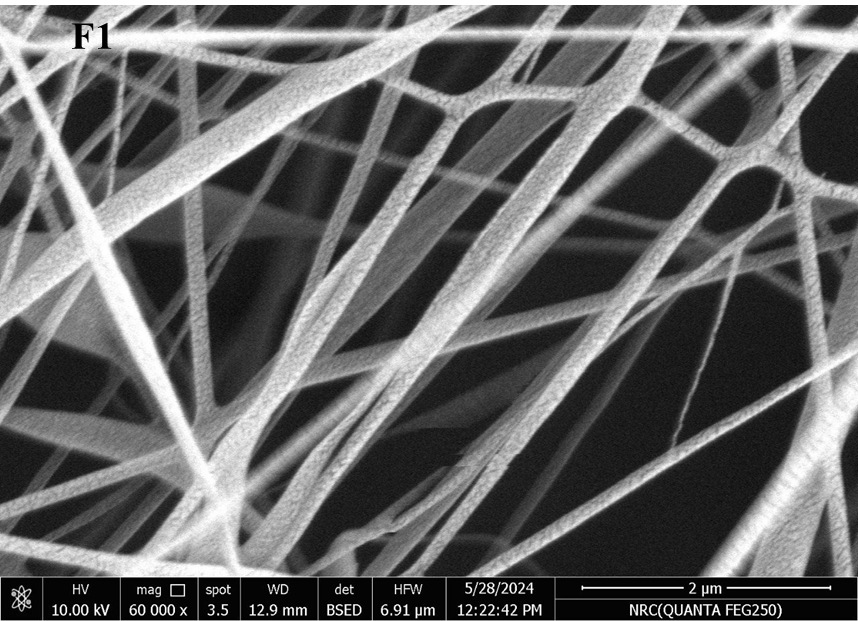


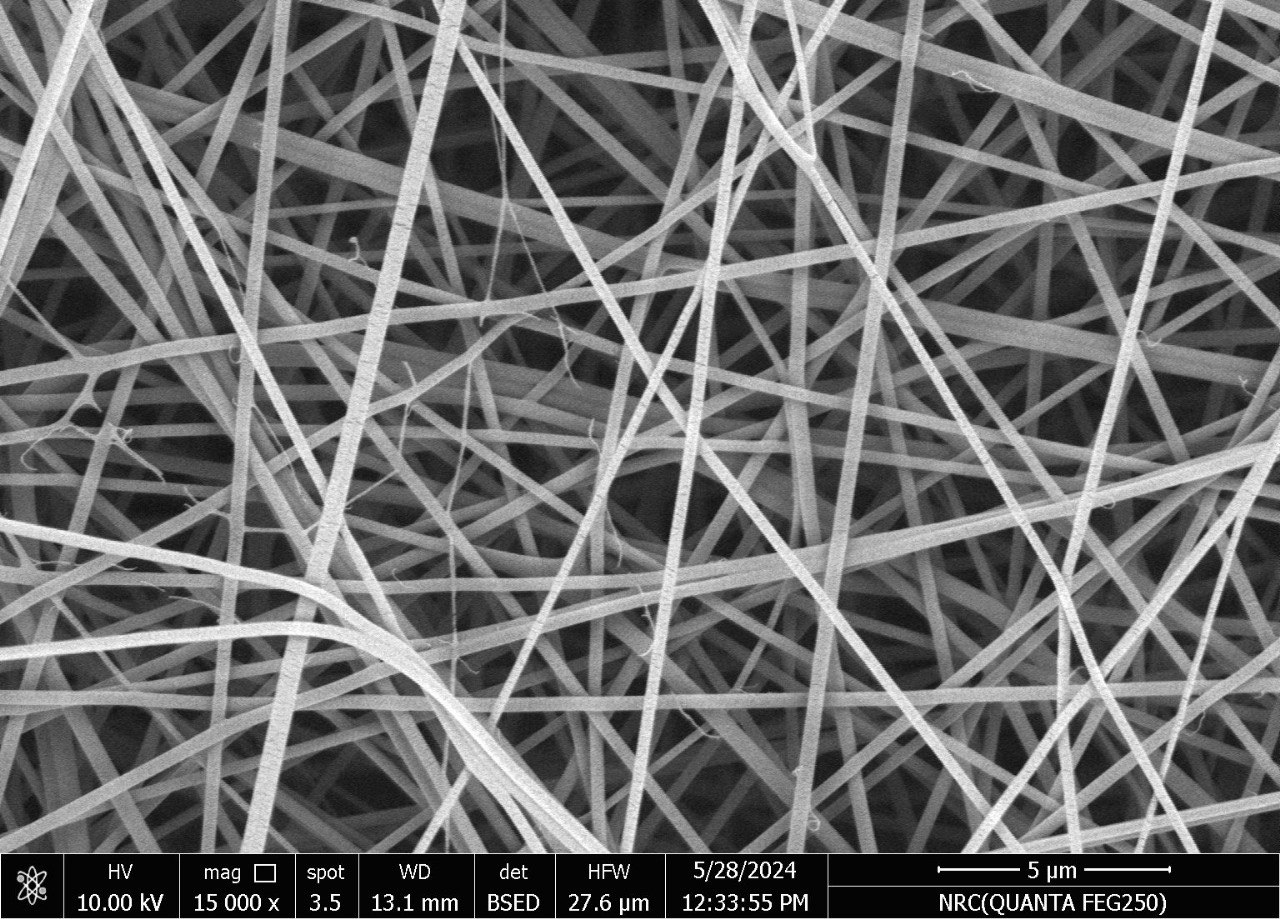


**F2**

**Supplementary Figure 1.** The SEM image of DOM-loaded EL-100/PVA NFs (F1 and F2 ).


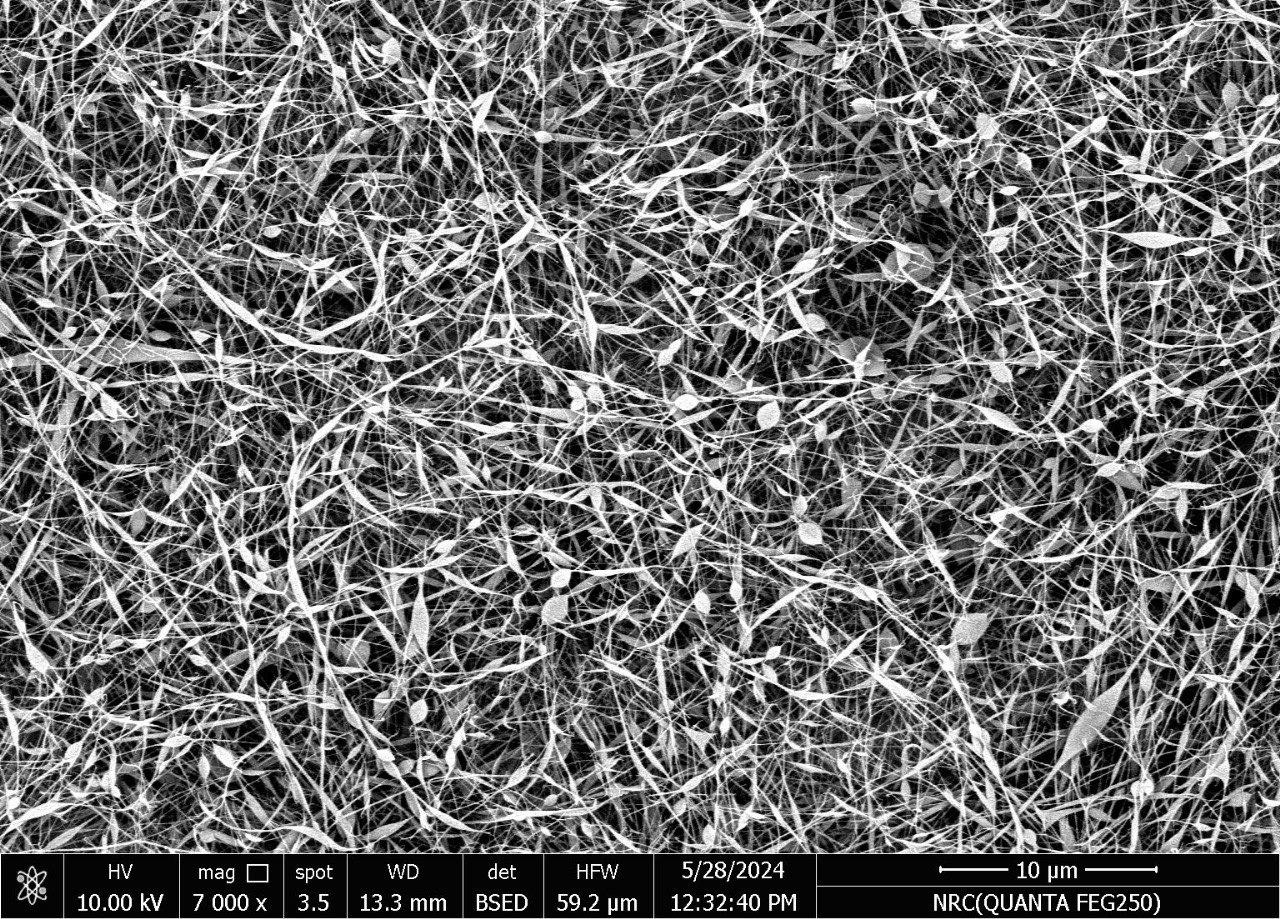


**Supplementary Figure 2.** The SEM image of DOM-loaded NFs prepared using EL-100/PVA NFs polymer blend of (40:60).


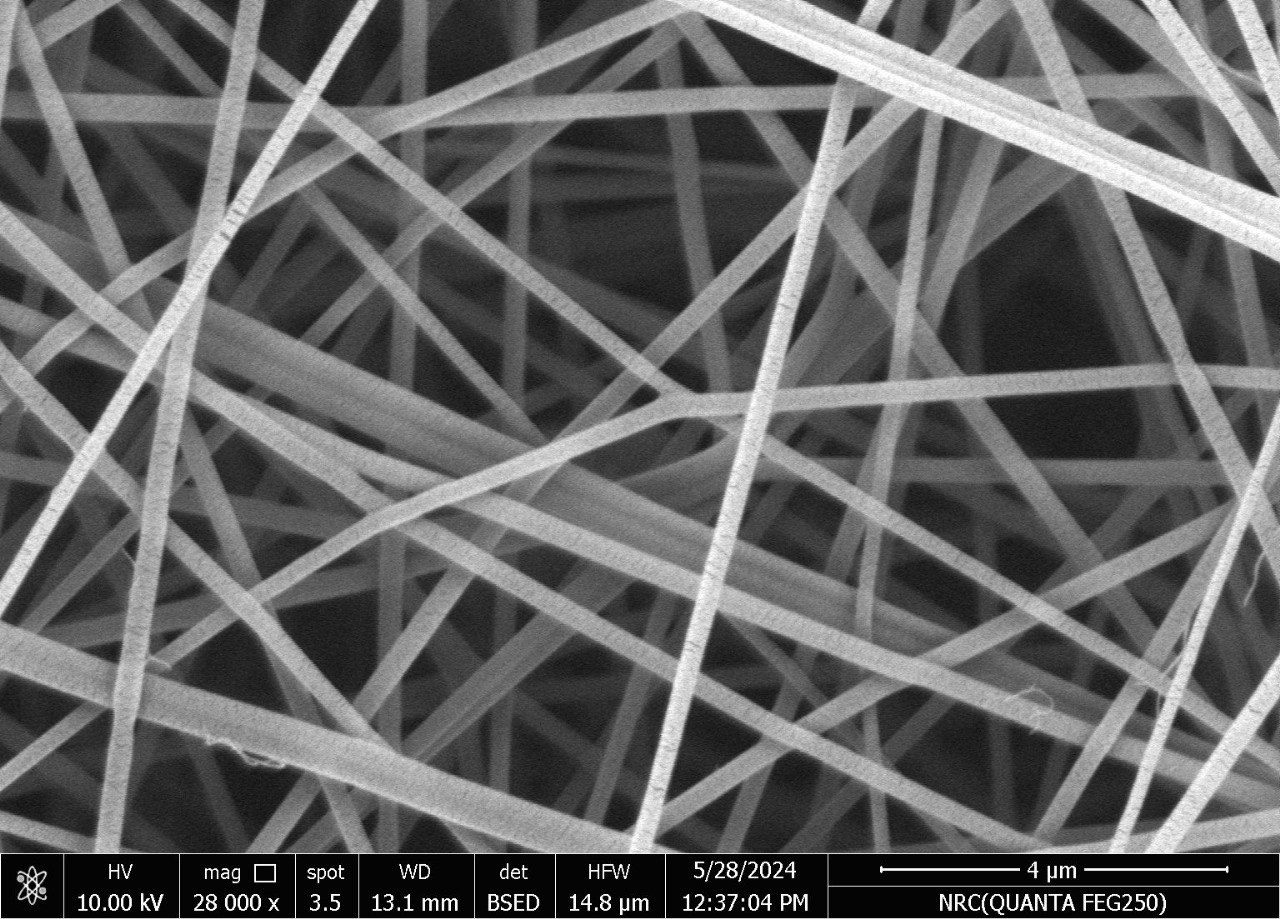


**F4**


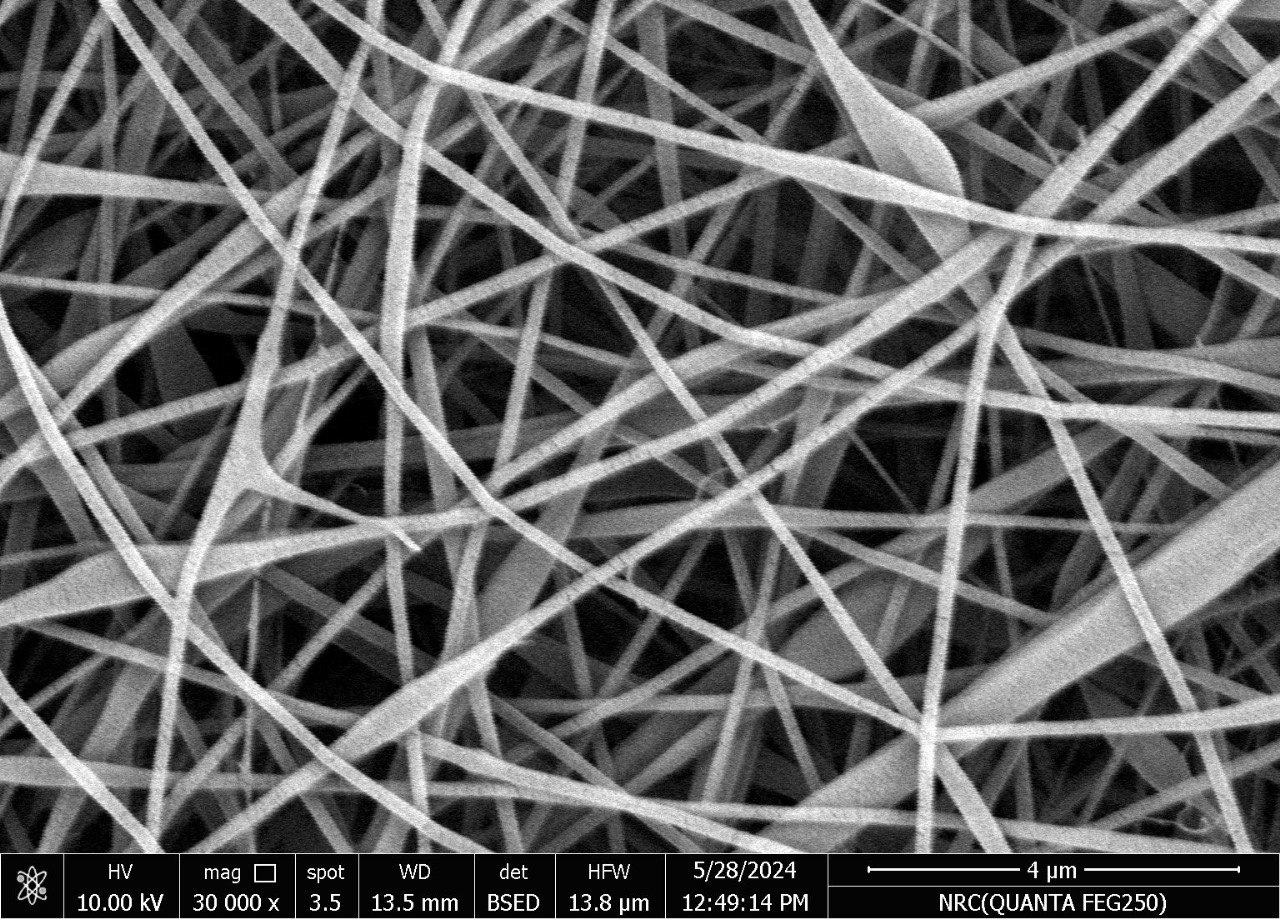


**F5**

**F4**

**Supplementary Figure 3.** The SEM image of DOM-loaded EL-100/PVA NFs (F4 and F5).


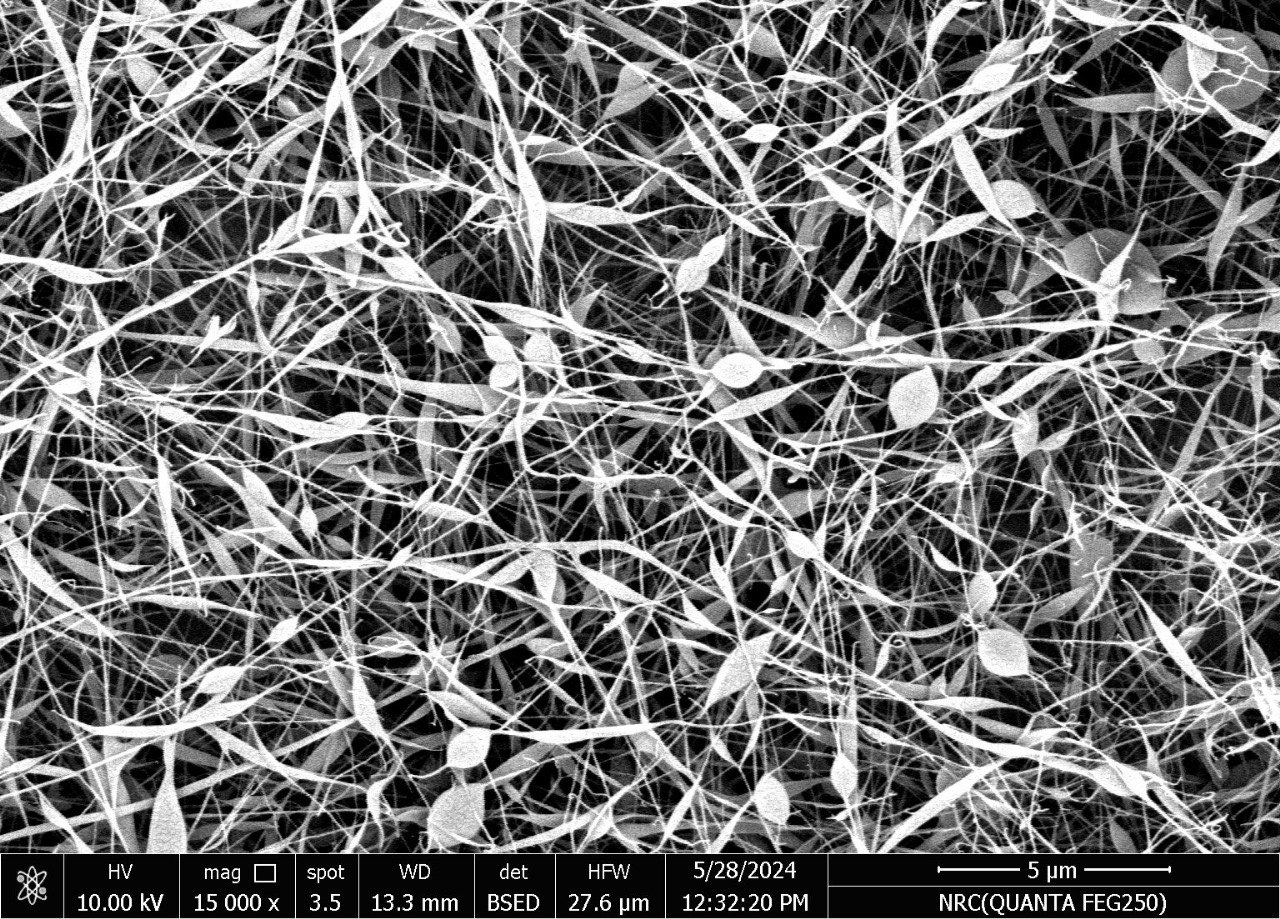


**F6**


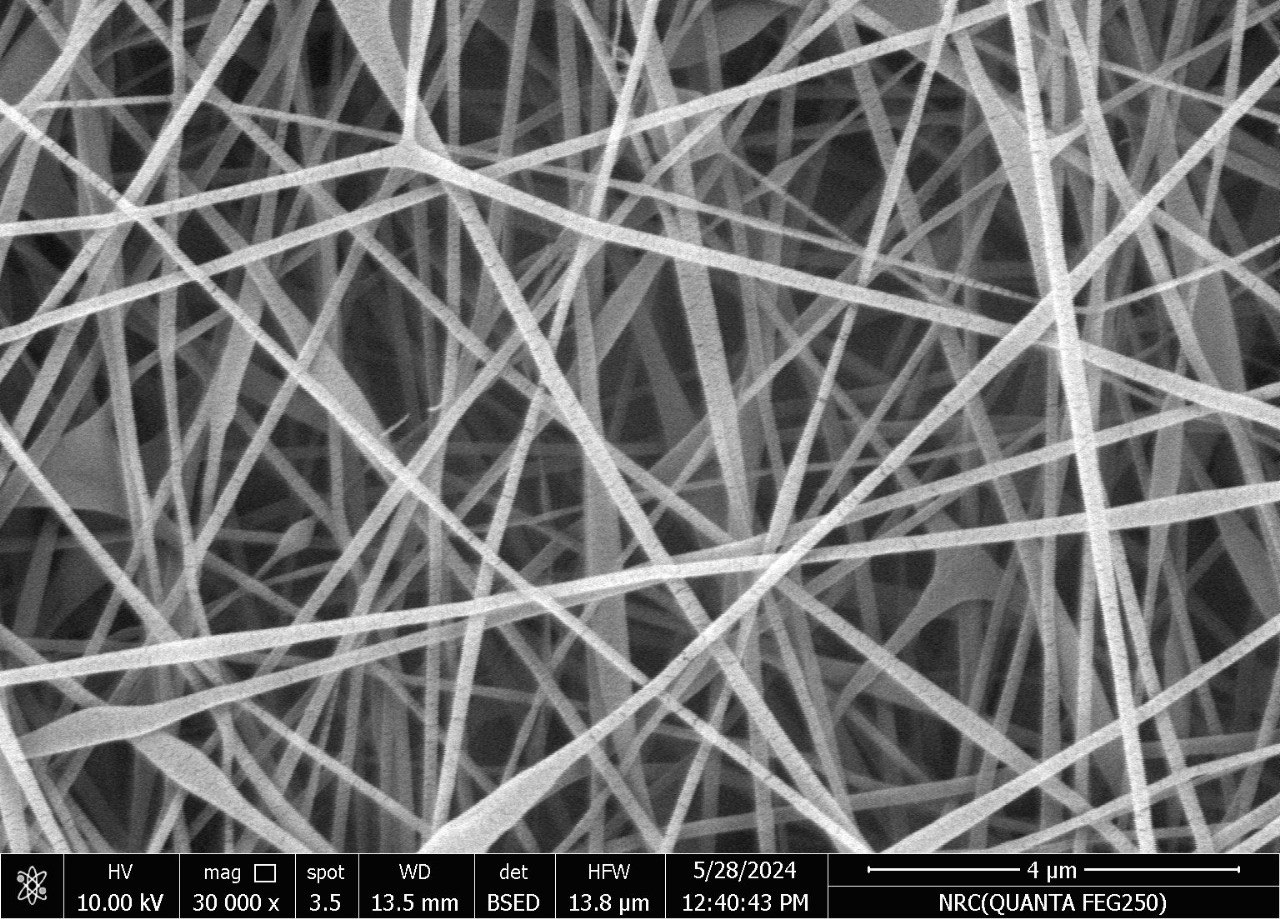


**F7**

**Supplementary Figure 4.** The SEM image of DOM-loaded EL-100/PVA NFs (F6 and F7).

**Supplementary Figure 5.** The *in vitro* release of free DOM and DOM-loaded NFs (F1 and F2) at a pH of 6.8. Results are Mean ± SD & n=3.

**Supplementary Figure 6.** The *in vitro* release of free DOM and DOM-loaded NFs (F4 and F5) at a pH of 6.8. Results are Mean ± SD & n=3.

**Supplementary Figure 7.** The *in vitro* release of free DOM and DOM-loaded NFs (F6 and F7) at a pH of 6.8. Results are Mean ± SD & n=3.

**Supplementary Table 1.** Drug release kinetic parameters of DOM-loaded EL-100/PVA NFs formulations.

| **Formula** | **Coefficients of Determination (R^2^)** | | | **Korsmeyer-Peppas** | |
| --- | --- | --- | --- | --- | --- |
|  | **Zero-Order** | **First-Order** | **Higuchi- Order** | **R^2^** | **Diffusional Exponent (n)** |
| **F1** | 0.9345 | 0.8889 | 0.9856 | 0.99 | 0.268 |
| **F2** | 0.8547 | 0.7972 | 0.9466 | 0.9813 | 0.2793 |
| **F3** | 0.6935 | 0.6488 | 0.8303 | 0.9179 | 0.279 |
| **F4** | 0.7837 | 0.7478 | 0.8906 | 0.9498 | 0.2547 |
| **F5** | 0.8342 | 0.7876 | 0.9262 | 0.9664 | 0.2877 |
| **F6** | 0.8973 | 0.8347 | 0.974 | 0.9949 | 0.2709 |
| **F7** | 0.7007 | 0.6903 | 0.8136 | 0.8982 | 0.2562 |
